# Supplementary material for: Integrating active and passive environmental DNA sampling into coral reef assessments: implications for community-based biomonitoring in Hawaiʻi
Source: BMC Zool. 2026 Oct 13;11:38. doi: 10.1186/s40850-026-00293-z (PMC13718060; doi:10.1186/s40850-026-00293-z)
Supplement: Supplementary file 1 [file 40850_2026_293_MOESM1_ESM.docx]

**Supplementary Information for**

Integrating active and passive environmental DNA sampling into coral reef assessments: implications for community-based biomonitoring in Hawaiʻi

Cécile M. Vimond^1*^, Zoe Hill^1^, Cameron A.J. Walsh^2^, Pamela Weiant^3^, Kēhaulani Kupihea^4^, Peter B. Marko^1,5^, Patrick K. Nichols^1,6*^

^1^School of Life Sciences, University of Hawaiʻi at Mānoa, Honolulu, HI, USA

^2^Hawaiʻi Institute of Marine Biology, School of Ocean and Earth Science and Technology, University of Hawai‘i at Mānoa, Kāneʻohe, HI, USA

^3^Mālama Maunalua, Honolulu, HI, USA

^4^Mauliola Keʻehi, Honolulu, HI, USA

^5^Oregon Institute of Marine Biology, University of Oregon, Charleston, OR, USA

^6^Ecology and Genetics Research Unit, University of Oulu, Oulu, Finland

*corresponding authors: cvimond@hawaii.edu; patrick.nichols@oulu.fi

**Figure S1.** (a) Proportion of eDNA contribution (based on eDNA index) across the three coral-specific metabarcoding markers (12S, 16S, ITS2) and visual percent cover for coral genera detected at the four temporal monitoring sites on Oʻahu, Hawaiʻi. (b) eDNA index contributions recovered through time across markers and sites. (c) Coefficient of variation (CV) of abundance-based signal intensity and (d) temporal detection frequency, calculated as the proportion of timepoints in which a genus was detected within each site for each marker. Points represent individual samples; boxplots and violin plots summarize distributions across markers. Letters denote post hoc pairwise comparison groups (shared letters indicate no significant difference; α = 0.05) within each respective model (LMM for CV, GLMM for detection frequency; see **Table S2** for full model results).

**Figure S2.** Overlap of detected zero-radius operational taxonomic units (zOTUs) between active and passive filtration methods across six taxonomic ranks (phylum through genus). The left bar chart shows the total number of zOTUs detected by each filtration method. The bottom dot matrix indicates membership: each vertical column represents a unique combination of methods (active, passive, or both); filled dots indicate which methods contribute to that combination. The top bar chart shows the number of zOTUs in each combination, with bars stacked and shaded by taxonomic rank to indicate the resolution of the overlapping zOTUs (phylum to genus).

**Figure S3.** Zeta diversity and core taxa by filtration method (active vs. passive). (a) Decline in shared zero-radius operational taxonomic units (zOTUs) and (b) retention ratio across zeta orders. (c) Relative eDNA read proportions for core families detected by filtration method. Proportions were calculated within each filtration method with stacked bars indicating contributions from each filtration method.

**Table S1.** Detection of scleractinian coral genera across sites using conventional visual benthic surveys and three coral-specific eDNA metabarcoding markers (12S, 16S, ITS2). Presence is indicated by “X”. Totals represent the number of genus detections per method across all sites.

| **Site** | **Genus** | **Visual** | **12S eDNA** | **16S eDNA** | **ITS2 eDNA** |
| --- | --- | --- | --- | --- | --- |
| Kaiona | *Porites spp.* | X | X | X | X |
|  | *Pocillopora spp.* | X | X | X | X |
|  | *Montipora spp.* | X | X | X | X |
|  | *Pavona spp.* | X | X | X | X |
|  | *Leptastrea spp.* | X | X | X | X |
|  | *Cyphastrea spp.* | X | X | X | X |
| Lanikai | *Porites spp.* | X | X | X | X |
|  | *Pocillopora spp.* | X | X | X | X |
|  | *Montipora spp.* | X | X | X | X |
|  | *Pavona spp.* |  | X | X | X |
|  | *Leptastrea spp.* |  | X | X |  |
|  | *Cyphastrea spp.* |  | X | X | X |
| Moku o Loʻe | *Porites spp.* | X | X | X | X |
|  | *Pocillopora spp.* | X | X | X | X |
|  | *Montipora spp.* | X | X | X | X |
|  | *Pavona spp.* |  |  |  | X |
|  | *Leptastrea spp.* | X | X | X | X |
|  | *Cyphastrea spp.* |  | X |  | X |
| Wailupe | *Porites spp.* | X | X | X | X |
|  | *Pocillopora spp.* | X | X | X | X |
|  | *Montipora spp.* | X | X | X | X |
|  | *Pavona spp.* |  | X | X | X |
|  | *Leptastrea spp.* | X | X | X | X |
|  | *Cyphastrea spp.* |  | X | X |  |
| ***Total*** | | ***17*** | ***23*** | ***22*** | ***22*** |

**Table S2.** Linear mixed-effects model (LMM) and generalized linear mixed-effects model (GLMM) results comparing temporal variability among coral eDNA markers. Two response variables were analyzed: (i) the coefficient of variation (CV) of abundance-based signal intensity, and (ii) detection frequency (incidence), modeled with binomial error structure. Marker identity was included as a fixed effect in both models, with site and genus included as random intercepts to account for spatial structure and repeated measures. Parameter estimates (β), standard errors (SE), t-values with degrees of freedom (df), z-values, and p-values are reported relative to the reference marker (12S).

| **Metric** | **Fixed effect** | **Estimate (β)** | **SE** | **z-value** | **t_df_** | **p** |
| --- | --- | --- | --- | --- | --- | --- |
| CV (LMM) | Intercept | -2.23 | 2.67 | - | -0.84_3.99_ | 0.45 |
|  | 16S | -0.20 | 0.19 | - | -1.05_56.2_ | 0.30 |
|  | ITS2 | 0.96 | 0.19 | - | 5.06_56.3_ | < 0.001 |
| Detection frequency (GLMM) | Intercept | -0.70 | 0.53 | -1.32 | - | 0.19 |
|  | 16S | 0.64 | 0.17 | 3.69 | - | < 0.001 |
|  | ITS2 | -1.83 | 0.10 | -19.1 | - | < 0.001 |

**Table S3.** Complete metazoan and limu (algae) species list detected across regions and markers.

**Table S4.** Taxa uniquely detected by active (2-L water filtration) versus passive eDNA collection for coral reef-associated metazoans and macroalgae. Taxa are organized by taxonomic rank (phylum to genus) and listed only if detected exclusively by one method across all samples.

| **Method** | **Rank** | **Unique taxa** |
| --- | --- | --- |
| Active | phyla | Xenacoelomorpha |
|  | classes | Acoela, Arachnida, Branchiopoda, Cephalopoda, Chondrichthyes, Leptocardii, Mammalia, Palaeonemertea, Thecostraca, Trematoda |
|  | orders | Acoela, Amphioxiformes, Archinemertea, Artiodactyla, Atheriniformes, Aulopiformes, Azygiida, Beloniformes, Blenniiformes, Characiformes, Cheilostomatida, Chondrillida, Cichliformes, Cladophorales, Coronatae, Cycloneritida, Cyprinodontiformes, Dendroceratida, Diplostraca, Ellobiida, Elopiformes, Holocentriformes, Istiophoriformes, Lophiiformes, Lutjaniformes, Mugiliformes, Myliobatiformes, Neogastropoda, Octopoda, Ophiurida, Pectinida, Perciformes, Polyarthra, Ralfsiales, Rhodachlyales, Sphacelariales, Testudines, Trochida, Trombidiformes |
|  | families | Acestrorhamphidae, Actiniidae, Aeolidiidae, Anchimolgidae, Anomiidae, Antennariidae, Aplysinidae, Atherinidae, Belonidae, Blenniidae, Boloceroididae, Branchiostomatidae, Caliphyllidae, Canuellidae, Caprellidae, Caulacanthaceae, Cephalotrichidae, Cheloniidae, Cichlidae, Cladonematidae, Cladophoraceae, Congridae, Corycaeidae, Corynidae, Delphinidae, Dictyodendrillidae, Discodorididae, Dorvilleidae, Dysideidae, Ectocarpaceae, Eleotridae, Ellobiidae, Elopidae, Epialtidae, Galaxauraceae, Gelidiellaceae, Gigartinaceae, Golfingiidae, Grapsidae, Gymnodorididae, Halacaridae, Heteropiidae, Hippolytidae, Hirudinellidae, Holocentridae, Hydractiniidae, Iotrochotidae, Irciniidae, Istiophoridae, Kallymeniaceae, Kirchenpaueriidae, Kyphosidae, Leucettidae, Lineidae, Linuchidae, Lutjanidae, Macrotrichidae, Mesosporaceae, Mithracidae, Mugilidae, Muricidae, Myliobatidae, Nephtheidae, Neritidae, Octopodidae, Oncaeidae, Ophiactidae, Orbiniidae, Palaemonidae, Pandeidae, Phloeodictyidae, Phyllodocidae, Planaxidae, Poeciliidae, Polynemidae, Polynoidae, Pomacentridae, Portunidae, Processidae, Protodrilidae, Pseudoceratinidae, Pyramidellidae, Rhizophyllidaceae, Rhodachlyaceae, Saccocirridae, Sagartiidae, Sagittiferidae, Scorpaenidae, Smaragdinellidae, Soleneiscidae, Sphacelariaceae, Spirostyliferinidae, Stichodactylidae, Stichopodidae, Sycettidae, Synaptidae, Synodontidae, Talitridae, Tetillidae, Thalassematidae, Thalestridae, Thoosidae, Thorectidae, Tripterygiidae, Tritoniidae, Trochidae, Watersiporidae, Xanthidae |
|  | genera | Abdopus, Abudefduf, Aetobatus, Aiptasia, Alectona, Amansia, Anchimolgus, Antennarius, Astyanax, Asymmetron, Atherinomorus, Atys, Bathygobius, Bestiolina, Biuve, Boloceroides, Botrylloides, Brachidontes, Breviturma, Cacospongia, Calliotrochus, Calotomus, Canthigaster, Caprella, Carminodoris, Caulacanthus, Centroceras, Cephalothrix, Ceratodictyon, Chaetonotus, Chelidonura, Chelonia, Chlorurus, Chondracanthus, Cinachyra, Cirripectes, Cladonema, Cladophora, Clathria, Claudrilus, Coeloplana, Coelothrix, Conger, Convolutriloba, Cosmocalanus, Crouania, Cupolaconcha, Cyerce, Dasysiphonia, Dendropoma, Diplomma, Dodecaceria, Dolabrifera, Dorvillea, Dysidea, Echinothrix, Ectenurus, Ectocarpus, Eleotris, Elops, Elysia, Enneapterygius, Entomacrodus, Eudactylopus, Eviota, Farranula, Fasciospongia, Galaxaura, Gelidiella, Gnatholepis, Gymnodoris, Gymnothorax, Herdmania, Hinea, Hippolyte, Hydractinia, Hydroclathrus, Hydrolithon, Igernella, Iotrochota, Ircinia, Jania, Kirchenpaueria, Kyphosus, Lamprohaminoea, Leodice, Leucandra, Linopherus, Linuche, Lissodendoryx, Lutjanus, Macrothrix, Malacoceros, Marionia, Meiodrilus, Melampus, Meredithia, Mesospora, Metopograpsus, Micippa, Monia, Monocorophium, Morula, Mugil, Myrianida, Myripristis, Naineris, Naso, Nematonereis, Neoniphon, Nephasoma, Nerilla, Nicidion, Nicolea, Nikoides, Oceanapia, Octopus, Odontosyllis, Oncaea, Ophiactis, Ophiocomella, Opisthosyllis, Osmundea, Osteomugil, Otohimella, Otopleura, Palisada, Pandeopsis, Paralepidonotus, Phanerophthalmus, Pharyngocirrus, Phyllodoce, Pilodius, Pione, Plakobranchus, Platybelone, Plectroglyphidodon, Pleurehdera, Poecilia, Polydactylus, Polydora, Polyplectana, Porphyridium, Portieria, Pristiapogon, Protodrilus, Pseudoceratina, Psilogobius, Pyura, Raphidrilus, Rhinecanthus, Rhodachlya, Rhombognathus, Rhyncholagena, Rosenvingea, Sargassum, Sargocentron, Sarotherodon, Saurida, Scarus, Scorpaenodes, Scottolana, Selar, Simocarcinus, Soleneiscus, Sphacelaria, Stelletta, Stenopeltis, Stereonephthya, Stethojulis, Stichopus, Stilophora, Styliferina, Sycettusa, Sycon, Syllidia, Syllis, Synodus, Terebella, Tetranemertes, Thalamita, Thelepus, Trochonerilla, Tursiops, Ululania, Uropterygius, Viatrix, Watersipora |
| Passive | phyla | Phaeophyceae |
|  | classes | Cubozoa |
|  | orders | Acrochaetiales, Carditida, Carybdeida, Macrodasyida, Monhysterida, Ostreoida, Palmariales, Siphonostomatoida, Trachymedusae |
|  | families | Acrochaetiaceae, Alatinidae, Carditidae, Clytiidae, Ectinosomatidae, Leptocytheridae, Opheliidae, Palmariaceae, Pennellidae, Rhodymeniaceae, Rhopalonematidae, Schindleriidae, Thaumastodermatidae, Xyalidae |
|  | genera | Acrochaetium, Aglaura, Alatina, Clytia, Daptonema, Diplodasys, Erythrocolon, Halichaetoderma, Lernaeenicus, Melanothamnus, Nannocalanus, Palmaria, Philinopsis, Polycarpa, Polyophthalmus, Schindleria, Tolypiocladia |

**Table S5.** Observed and extrapolated asymptotic richness of zero-radius operational taxonomic units (zOTUs) across regions (Keʻehi and Maunalua) and eDNA collection methods (active vs. passive) for each genetic marker: algae (23S), corals/sponges (16S), total eukaryotes (18S), fish (MFU), and macroinvertebrates (COI). Extrapolated richness estimates are presented as mean ± standard error (SE).

| **Marker** | **Grouping** | **Observed Richness** | **Extrapolated (± SE)** |
| --- | --- | --- | --- |
| Algae (23S) | Overall | 127 | 148 ± 12 |
|  | Active | 92 | 120 ± 15 |
|  | Passive | 109 | 176 ± 27 |
|  | Keʻehi | 61 | 91 ± 13 |
|  | Maunalua | 90 | 98 ± 8 |
| Corals/sponges (16S) | Overall | 31 | 32 ± 1 |
|  | Active | 31 | 32 ± 1 |
|  | Passive | 23 | 47 ± 17 |
|  | Keʻehi | 19 | 23 ± 7 |
|  | Maunalua | 31 | 32 ± 2 |
| Eukaryotes (18S) | Overall | 1049 | 1307 ± 34 |
|  | Active | 778 | 963 ± 32 |
|  | Passive | 786 | 1089 ± 36 |
|  | Keʻehi | 560 | 796 ± 33 |
|  | Maunalua | 699 | 790 ± 18 |
| Fish (MFU) | Overall | 118 | 144 ± 12 |
|  | Active | 114 | 131 ± 10 |
|  | Passive | 36 | 70 ± 16 |
|  | Keʻehi | 35 | 52 ± 14 |
|  | Maunalua | 112 | 140 ± 11 |
| Inverts (COI) | Overall | 1164 | 1831 ± 67 |
|  | Active | 1015 | 1477 ± 52 |
|  | Passive | 328 | 884 ± 97 |
|  | Keʻehi | 365 | 998 ± 93 |
|  | Maunalua | 934 | 1312 ± 51 |

**Table S6.** Significant indicator taxa associated with each embayment region (Keʻehi and Maunalua Bay) based on indicator species analysis of eDNA metabarcoding data. Taxa are listed with their corresponding genetic marker and indicator value (stat). P-values (p.val) and multiple testing-corrected p-values (p.adj; Benjamini-Hochberg) are reported.

| **Region** | **Taxa** | **Marker** | **stat** | **p.val** | **p.adj** |
| --- | --- | --- | --- | --- | --- |
| Keʻehi | Capitellidae (Annelida) | Eukaryotes (18S) | 0.422 | <0.001 | 0.005 |
|  | Poritidae (Cnidaria) | Eukaryotes (18S) | 0.337 | <0.001 | 0.005 |
|  | Chaetopteridae (Annelida) | Eukaryotes (18S) | 0.298 | 0.001 | 0.010 |
|  | Aplysiidae (Mollusca) | Eukaryotes (18S) | 0.288 | 0.001 | 0.013 |
|  | Amphinomidae (Annelida) | Eukaryotes (18S) | 0.283 | 0.001 | 0.013 |
|  | Ostreidae (Mollusca) | Eukaryotes (18S) | 0.273 | <0.001 | 0.005 |
|  | Limidae (Mollusca) | Eukaryotes (18S) | 0.263 | 0.001 | 0.013 |
|  | Ostreidae (Mollusca) | Inverts (COI) | 0.260 | <0.001 | 0.018 |
|  | Centropagidae (Arthropoda) | Eukaryotes (18S) | 0.233 | 0.005 | 0.036 |
|  | Pteriidae (Mollusca) | Eukaryotes (18S) | 0.217 | 0.006 | 0.039 |
| Maunalua | Rhodomelaceae (Rhodophyta) | Eukaryotes (18S) | 0.419 | <0.001 | 0.005 |
|  | Ceramiaceae (Rhodophyta) | Eukaryotes (18S) | 0.403 | <0.001 | 0.005 |
|  | Phaeophilaceae (Chlorophyta) | Eukaryotes (18S) | 0.375 | 0.001 | 0.011 |
|  | Peyssonneliaceae (Rhodophyta) | Eukaryotes (18S) | 0.361 | <0.001 | 0.007 |
|  | Corallinaceae (Rhodophyta) | Eukaryotes (18S) | 0.349 | 0.001 | 0.013 |
|  | Styelidae (Chordata) | Eukaryotes (18S) | 0.345 | <0.001 | 0.005 |
|  | Eunicidae (Annelida) | Eukaryotes (18S) | 0.345 | 0.001 | 0.013 |
|  | Ulvellaceae (Chlorophyta) | Eukaryotes (18S) | 0.344 | 0.001 | 0.013 |
|  | Ostreobiaceae (Chlorophyta) | Algae (23S) | 0.338 | 0.001 | 0.042 |
|  | Polyphysaceae (Chlorophyta) | Eukaryotes (18S) | 0.322 | 0.001 | 0.013 |
|  | Cirratulidae (Annelida) | Eukaryotes (18S) | 0.318 | 0.001 | 0.013 |
|  | Perophoridae (Chordata) | Eukaryotes (18S) | 0.308 | 0.002 | 0.017 |
|  | Callyspongiidae (Porifera) | Eukaryotes (18S) | 0.307 | 0.002 | 0.019 |
|  | Leucettidae (Porifera) | Eukaryotes (18S) | 0.305 | 0.002 | 0.019 |
|  | Mycalidae (Porifera) | Eukaryotes (18S) | 0.301 | 0.002 | 0.019 |
|  | Grantiidae (Porifera) | Eukaryotes (18S) | 0.280 | 0.003 | 0.020 |
|  | Lithophyllaceae (Rhodophyta) | Eukaryotes (18S) | 0.273 | 0.006 | 0.040 |
|  | Tribonemataceae (Ochrophyta) | Eukaryotes (18S) | 0.254 | 0.008 | 0.046 |
|  | Ulvaceae (Chlorophyta) | Eukaryotes (18S) | 0.252 | 0.005 | 0.036 |

**Table S7.** Significant indicator taxa associated with eDNA sampling method (active vs. passive) based on indicator species analysis. Taxa are listed with their corresponding genetic marker and indicator value (stat). P-values (p.val) and multiple testing-corrected p-values (p.adj; Benjamini-Hochberg) are reported.

| **Method** | **Taxa** | **Marker** | **stat** | **p.value** | **p.adjusted** |
| --- | --- | --- | --- | --- | --- |
| Active | Chalinidae (Porifera) | Eukaryotes (18S) | 0.524 | <0.001 | 0.001 |
|  | Phaeophilaceae (Chlorophyta) | Eukaryotes (18S) | 0.455 | <0.001 | 0.001 |
|  | Leucettidae (Porifera) | Eukaryotes (18S) | 0.416 | <0.001 | 0.001 |
|  | Peyssonneliaceae (Rhodophyta) | Eukaryotes (18S) | 0.414 | <0.001 | 0.001 |
|  | Poritidae (Cnidaria) | Corals/Sponges (16S) | 0.405 | <0.001 | 0.001 |
|  | Sagittiferidae (Xenacoelomorpha) | Inverts (COI) | 0.404 | <0.001 | 0.001 |
|  | Chalinidae (Porifera) | Inverts (COI) | 0.39 | <0.001 | 0.001 |
|  | Clionaidae (Porifera) | Inverts (COI) | 0.387 | <0.001 | 0.001 |
|  | Polyphysaceae (Chlorophyta) | Inverts (COI) | 0.377 | <0.001 | 0.001 |
|  | Microcionidae (Porifera) | Inverts (COI) | 0.372 | <0.001 | 0.001 |
|  | Holothuriidae (Echinodermata) | Inverts (COI) | 0.363 | <0.001 | 0.002 |
|  | Halichondriidae (Porifera) | Inverts (COI) | 0.361 | <0.001 | 0.001 |
|  | Callyspongiidae (Porifera) | Eukaryotes (18S) | 0.349 | <0.001 | 0.001 |
|  | Corallinaceae (Rhodophyta) | Eukaryotes (18S) | 0.347 | <0.001 | 0.003 |
|  | Dictyodendrillidae (Porifera) | Eukaryotes (18S) | 0.34 | <0.001 | 0.001 |
|  | Boloceroididae (Cnidaria) | Eukaryotes (18S) | 0.339 | <0.001 | 0.001 |
|  | Orbiniidae (Annelida) | Inverts (COI) | 0.339 | <0.001 | 0.001 |
|  | Cirratulidae (Annelida) | Eukaryotes (18S) | 0.334 | <0.001 | 0.001 |
|  | Heteropiidae (Porifera) | Eukaryotes (18S) | 0.334 | <0.001 | 0.001 |
|  | Eunicidae (Annelida) | Inverts (COI) | 0.332 | <0.001 | 0.001 |
|  | Echinometridae (Echinodermata) | Inverts (COI) | 0.327 | <0.001 | 0.001 |
|  | Coelosphaeridae (Porifera) | Inverts (COI) | 0.326 | <0.001 | 0.001 |
|  | Halichondriidae (Porifera) | Eukaryotes (18S) | 0.321 | 0.001 | 0.011 |
|  | Tedaniidae (Porifera) | Inverts (COI) | 0.321 | <0.001 | 0.001 |
|  | Grantiidae (Porifera) | Eukaryotes (18S) | 0.32 | <0.001 | 0.001 |
|  | Stichodactylidae (Cnidaria) | Eukaryotes (18S) | 0.319 | <0.001 | 0.001 |
|  | Syllidae (Annelida) | Inverts (COI) | 0.319 | <0.001 | 0.002 |
|  | Rhodomelaceae (Rhodophyta) | Inverts (COI) | 0.315 | 0.003 | 0.016 |
|  | Labridae (Chordata) | Fish (MFU) | 0.315 | 0.001 | 0.014 |
|  | Aplysinidae (Porifera) | Eukaryotes (18S) | 0.314 | <0.001 | 0.001 |
|  | Chordariaceae (Ochrophyta) | Inverts (COI) | 0.31 | <0.001 | 0.001 |
|  | Scytosiphonaceae (Ochrophyta) | Algae (23S) | 0.307 | 0.001 | 0.017 |
|  | Cerithiidae (Mollusca) | Inverts (COI) | 0.307 | 0.001 | 0.007 |
|  | Eunicidae (Annelida) | Eukaryotes (18S) | 0.305 | 0.002 | 0.013 |
|  | Haminoeidae (Mollusca) | Inverts (COI) | 0.304 | <0.001 | 0.001 |
|  | Scytosiphonaceae (Ochrophyta) | Inverts (COI) | 0.303 | 0.001 | 0.005 |
|  | Plakobranchidae (Mollusca) | Inverts (COI) | 0.302 | <0.001 | 0.001 |
|  | Mycalidae (Porifera) | Inverts (COI) | 0.299 | <0.001 | 0.001 |
|  | Portunidae (Arthropoda) | Inverts (COI) | 0.299 | <0.001 | 0.001 |
|  | Sycettidae (Porifera) | Eukaryotes (18S) | 0.296 | <0.001 | 0.001 |
|  | Soleneiscidae (Porifera) | Eukaryotes (18S) | 0.294 | <0.001 | 0.001 |
|  | Boloceroididae (Cnidaria) | Inverts (COI) | 0.294 | <0.001 | 0.001 |
|  | Terebellidae (Mollusca) | Inverts (COI) | 0.294 | 0.003 | 0.014 |
|  | Pomacentridae (Chordata) | Fish (MFU) | 0.287 | <0.001 | 0.007 |
|  | Tethyidae (Porites) | Inverts (COI) | 0.285 | 0.002 | 0.009 |
|  | Phloeodictyidae (Porifera) | Eukaryotes (18S) | 0.281 | <0.001 | 0.001 |
|  | Mycalidae (Porifera) | Eukaryotes (18S) | 0.278 | 0.004 | 0.026 |
|  | Corallinaceae (Rhodophyta) | Inverts (COI) | 0.274 | <0.001 | 0.001 |
|  | Leptastreidae (Cnidaria) | Inverts (COI) | 0.273 | 0.001 | 0.007 |
|  | Phyllodocidae (Annelida) | Eukaryotes (18S) | 0.27 | 0.001 | 0.006 |
|  | Dictyotaceae (Ochrophyta) | Inverts (COI) | 0.268 | 0.001 | 0.005 |
|  | Cladophoraceae (Chlorophyta) | Eukaryotes (18S) | 0.261 | 0.002 | 0.013 |
|  | Caliphyllidae (Mollusca) | Inverts (COI) | 0.254 | 0.014 | 0.048 |
|  | Aiptasiidae (Cnidaria) | Inverts (COI) | 0.252 | <0.001 | 0.001 |
|  | Mullidae (Chordata) | Fish (MFU) | 0.249 | 0.003 | 0.025 |
|  | Irciniidae (Porifera) | Corals/Sponges (16S) | 0.245 | <0.001 | 0.002 |
|  | Holocentridae (Chordata) | Fish (MFU) | 0.241 | <0.001 | 0.005 |
|  | Thorectidae (Porifera) | Inverts (COI) | 0.24 | 0.011 | 0.042 |
|  | Protodrilidae (Annelida) | Inverts (COI) | 0.239 | 0.005 | 0.022 |
|  | Kyphosidae (Chordata) | Fish (MFU) | 0.239 | 0.002 | 0.020 |
|  | Thalassematidae (Annelida) | Eukaryotes (18S) | 0.237 | 0.002 | 0.012 |
|  | Hypneaceae (Rhodophyta) | Algae (23S) | 0.237 | 0.002 | 0.017 |
|  | Gymnodorididae (Mollusca) | Inverts (COI) | 0.234 | 0.005 | 0.023 |
|  | Octopodidae (Mollusca) | Inverts (COI) | 0.232 | 0.005 | 0.022 |
|  | Callyspongiidae (Porifera) | Inverts (COI) | 0.232 | 0.001 | 0.005 |
|  | Cirratulidae (Annelida) | Inverts (COI) | 0.222 | 0.005 | 0.022 |
|  | Gelidiellaceae (Rhodophyta) | Inverts (COI) | 0.22 | 0.012 | 0.042 |
|  | Ampithoidae (Arthropoda) | Inverts (COI) | 0.215 | 0.002 | 0.009 |
|  | Halichondriidae (Porifera) | Corals/Sponges (16S) | 0.214 | 0.003 | 0.009 |
|  | Dasyaceae (Rhodophyta) | Inverts (COI) | 0.213 | 0.014 | 0.048 |
|  | Thalestridae (Arthropoda) | Eukaryotes (18S) | 0.212 | 0.004 | 0.026 |
|  | Kyphosidae (Chordata) | Inverts (COI) | 0.208 | 0.01 | 0.04 |
|  | Dorvilleidae (Annelida) | Inverts (COI) | 0.207 | <0.001 | 0.002 |
|  | Styelidae (Chordata) | Inverts (COI) | 0.206 | 0.01 | 0.04 |
|  | Spirastrellidae (Porifera) | Inverts (COI) | 0.205 | <0.001 | 0.003 |
|  | Mycalidae (Porifera) | Corals/Sponges (16S) | 0.204 | 0.006 | 0.017 |
|  | Oscarellidae (Porifera) | Inverts (COI) | 0.204 | <0.001 | 0.002 |
|  | Smaragdinellidae (Mollusca) | Inverts (COI) | 0.19 | 0.005 | 0.022 |
|  | Poseidonemertidae (Nemertea) | Inverts (COI) | 0.188 | 0.004 | 0.019 |
|  | Ascidiidae (Chordata) | Inverts (COI) | 0.186 | 0.011 | 0.042 |
|  | Plakinidae (Porifera) | Eukaryotes (18S) | 0.177 | 0.005 | 0.032 |
|  | Golfingiidae (Annelida) | Inverts (COI) | 0.171 | 0.005 | 0.022 |
|  | Sphacelariaceae (Ochrophyta) | Algae (23S) | 0.165 | 0.005 | 0.036 |
|  | Cichlidae (Chordata) | Fish (MFU) | 0.164 | 0.002 | 0.020 |
|  | Galaxauraceae (Rhodophyta) | Inverts (COI) | 0.153 | 0.006 | 0.024 |
|  | Canuellidae (Arthropoda) | Eukaryotes (18S) | 0.151 | 0.002 | 0.013 |
|  | Pocilloporidae (Cnidaria) | Eukaryotes (18S) | 0.147 | <0.001 | 0.001 |
|  | Sagartiidae (Cnidaria) | Inverts (COI) | 0.147 | 0.013 | 0.047 |
|  | Lomentariaceae (Rhodophyta) | Inverts (COI) | 0.145 | 0.006 | 0.025 |
|  | Aiptasiidae (Cnidaria) | Corals/Sponges (16S) | 0.144 | <0.001 | 0.001 |
|  | Corynidae (Cnidaria) | Inverts (COI) | 0.139 | 0.009 | 0.036 |
| Passive | Clausocalanidae (Arthropoda) | Inverts (COI) | 0.244 | 0.013 | 0.046 |
